# RubberFormer: a transformer-based detection benchmark for rubber tree powdery mildew

**DOI:** 10.3389/fpls.2026.1836334

**Published:** 2026-06-10

**Authors:** Yuheng Li, Jiazheng Zhu, Xilong Zhu, Lijuan Zhou, Qian Chen, Yu Zhang

**Affiliations:** 1 School of Cyberspace Security (School of Cryptology), Hainan University, Haikou, China; 2 Key Laboratory of Internet Information Retrieval of Hainan Province, Haikou, China; 3 School of Tropical Agriculture and Forestry, Hainan University, Danzhou, China; 4 Sanya Institute of Breeding and Multiplication, Hainan University, Sanya, China

**Keywords:** agricultural visual recognition, cross-dimensional attention, rubber tree powdery mildew, small object detection, transformer-based detection

## Abstract

**Introduction:**

Rubber tree powdery mildew is a major foliar disease that threatens the yield and quality of natural rubber. Its lesions are typically small, irregular, and embedded in complex backgrounds, making accurate automated detection difficult.

**Methods:**

To address this challenge, we propose RubberFormer, an end-to-end detection framework based on a refined Transformer architecture for detecting small powdery mildew lesions in complex scenarios. RubberFormer adopts MobileNetV4 as a lightweight backbone, introduces the Hierarchical Attention with Local-global Optimization (HALO) module for multiscale local-global feature fusion, incorporates the Unified Cross-Attention Network (UCAN) to enhance multidimensional feature interaction, and applies Normalized Wasserstein Distance (NWD) Loss to improve small-object localization.

**Results:**

Extensive experiments were conducted on PM-Dataset-Plus, which contains 9,765 images, and PD-40, a large-scale plant disease dataset containing 80,369 images across 40 disease categories and 8 crops. RubberFormer achieved superior detection accuracy and generalization performance compared with existing methods, while maintaining computational efficiency suitable for practical agricultural monitoring.

**Discussion:**

These results demonstrate that RubberFormer is effective for detecting small and irregular rubber tree powdery mildew lesions under complex conditions. The framework has practical value for rubber tree disease monitoring and provides a transferable design strategy for agricultural vision tasks involving small objects and complex backgrounds.

## 1 Introduction

The rubber tree (*Hevea brasiliensis*) is a major tropical cash crop, and its latex serves as the primary source of natural rubber, which is widely utilized across industries such as manufacturing, agriculture, and medicine (Van Beilen and Poirier, 2007). However, powdery mildew, a prevalent foliar disease affecting rubber trees, impairs photosynthesis and causes nutrient loss; in severe cases, it can reduce rubber yield by over 30%, posing a substantial threat to the industry’s economic viability (Liyanage et al., 2016). Traditional detection methods for powdery mildew rely on manual inspection, which is time-consuming, labor-intensive, and subjective. Moreover, such methods are inadequate for large-scale and real-time monitoring, failing to meet the demands of precision agriculture. Current chemical control strategies, primarily involving the application of sulfur powder, also suffer from limitations, including poorly timed application windows and low control efficiency (Wang et al., 2014). Therefore, developing efficient and accurate automated detection technologies is crucial for early disease warning and precise management.

In recent years, deep learning techniques, particularly object detection methods based on convolutional neural networks (CNNs) (LeCun et al., 2002), have achieved remarkable advancements in plant disease identification. However, applying these models to real-world scenarios of rubber tree powdery mildew detection presents three major challenges. First, the disease spots are typically tiny (mostly less than 5 mm in diameter) and irregular in shape, making them easily confused with natural leaf textures, insect bite marks, and other background elements. Second, field environments are highly complex, with factors such as variable lighting and occlusion from branches and leaves, which increase the difficulty of feature extraction. Third, most existing models prioritize detection accuracy without adequately considering the deployment constraints of edge devices, limiting their applicability to mobile and real-time detection tasks.

To address the aforementioned challenges, the Transformer architecture (Ashish, 2017) has been introduced into object detection due to its powerful global modeling capabilities. Vision Transformer (ViT) (Dosovitskiy et al., 2021) demonstrated that pure Transformer models can achieve excellent results on image recognition tasks, while Swin Transformer (Liu et al., 2021) further improved efficiency through hierarchical feature maps and shifted window mechanisms. For example, the RT-DETR model (Zhao et al., 2024) achieves a balance between high accuracy and fast inference through an efficient attention mechanism. However, directly applying such general-purpose detection frameworks to rubber tree powdery mildew remains problematic. On the one hand, the standard Transformer has high computational complexity, which hampers deployment on resource-constrained edge devices. On the other hand, existing feature fusion strategies for small targets in complex backgrounds remain suboptimal.

To overcome these limitations, this paper proposes RubberFormer, an end-to-end detection framework based on an improved Transformer architecture, tailored for the automated detection of rubber tree powdery mildew. This framework addresses the identified issues through three key innovations. First, it utilizes a lightweight backbone network, MobileNetV4 (Qin et al., 2024), which reduces computational overhead while maintaining strong feature extraction performance, thereby meeting the requirements of edge deployment. Second, it introduces a *Hierarchical Attention Layered Optimization (HALO)* module that enhances multiscale spot recognition by fusing local and global information. Third, it develops a *Unified Cross Attention Network (UCAN)* combined with the *Normalized Wasserstein Distance (NWD)* Loss (Wang et al., 2021) to improve feature modeling and localization accuracy for small and irregular targets.

In addition, to verify the effectiveness of the proposed method, this study expanded the self-constructed rubber tree powdery mildew dataset, PM-Dataset (Li et al., 2024), from 6,200 to 9,765 images, resulting in PM-Dataset-Plus, and conducted comparative experiments on both this dataset and the PD-40 dataset (Li et al., 2025). Experimental results demonstrate that RubberFormer outperforms existing mainstream methods in terms of detection accuracy, speed, and generalization capability, offering a practical technical solution for real-time monitoring of rubber tree powdery mildew.

The main contributions of this study are summarized as follows:

1. We propose a novel detection framework, RubberFormer, which is based on an improved Transformer architecture. It integrates MobileNetV4 as the backbone network, achieving a balance between detection accuracy and model lightweighting, and is suitable for deployment on edge devices.
2. A hierarchical attention module, HALO, with local-global optimization is designed to perform adaptive fusion of multiscale features through a local-global attention mechanism, thereby improving the model’s feature representation capability in complex backgrounds.
3. UCAN is proposed to enhance the detection of small, morphologically irregular lesions. At the same time, NWD Loss is incorporated to mitigate the dependence of small-scale lesion detection on the intersection-over-union (IoU) metric.
4. The rubber tree powdery mildew dataset, PM-Dataset-Plus, was constructed by expanding the number of images from 6,200 to 9,765, and comprehensive experiments were conducted on this dataset as well as the PD-40 dataset.

## 2 Related work

Traditional approaches to plant disease detection primarily rely on manual inspection and expert judgment, often supplemented by image processing techniques that analyze low-level features, such as leaf color, texture, and edges (Zhang et al., 2012; Zeng et al., 2023b; Li et al., 2020). These methods played a crucial role in early research, utilizing techniques such as color thresholding and edge detection to localize diseased areas (Zeng et al., 2023a; Cheng et al., 2024). However, rubber tree powdery mildew in natural environments presents challenges, including tiny lesion size, blurred boundaries, and irregular morphology. These characteristics make traditional methods susceptible to variations in lighting and background noise, resulting in limited detection accuracy and poor robustness. Moreover, the low degree of automation restricts their applicability to large-scale, real-time monitoring in practical plantation scenarios.

With the rapid advancement of deep learning technologies, particularly CNNs, plant disease detection has progressively shifted from traditional methods based on manual feature extraction to end-to-end automatic recognition models (Mustofa et al., 2023; Zeng et al., 2024). Classical architectures such as AlexNet (Krizhevsky et al., 2012), VGG (Simonyan and Zisserman, 2014), and ResNet (He et al., 2016) have been widely applied to the classification and localization of crop leaf images. EfficientNet (Tan and Le, 2019) introduced compound scaling to balance network depth, width, and resolution, achieving state-of-the-art accuracy with improved efficiency. Building on this foundation, efficient object detection frameworks have been introduced into plant disease recognition. Among two-stage detectors, Faster R-CNN (Ren et al., 2015) remains a representative method that generates region proposals via a Region Proposal Network (RPN) and refines them through a dedicated classification and regression head. While Faster R-CNN achieves strong localization accuracy due to its explicit proposal refinement stage, its two-pass architecture incurs higher computational costs and limits real-time applicability. In the context of plant disease detection, Fuentes et al (Fuentes et al., 2017). applied Faster R-CNN with feature fusion for tomato disease recognition, achieving high precision but at the cost of slower inference speeds. In contrast, one-stage detectors such as the YOLO series (Ali and Zhang, 2024; Redmon et al., 2016) and SSD (Liu et al., 2016) directly predict bounding boxes and class labels in a single forward pass, offering significantly faster inference. Feature Pyramid Networks (FPN) (Lin et al., 2017a) and Path Aggregation Networks (PANet) (Liu et al., 2018) further enhanced multiscale feature fusion capabilities, enabling better detection of objects at various scales. Borhani et al. (2022). proposed a lightweight Vision Transformer approach for automated plant disease classification, demonstrating the potential of Transformer-based methods in agricultural applications. Zeng et al. (2022). proposed GMA-Net, which integrates multiscale inflationary convolution with a cross-scale attention mechanism to recognize rubber tree leaf diseases, achieving a classification accuracy of 98.06% on a self-constructed dataset. Li et al. (2024) developed PM-YOLO for the automatic detection and classification of rubber tree powdery mildew by incorporating a feature fusion and size-aware module, reaching a mean average precision (mAP) of 86.9%, outperforming YOLOv10. Yang et al. (2023). introduced LS-YOLOv8s, based on YOLOv8 and featuring an improved feature pyramid and loss function, which enhanced detection accuracy and robustness for strawberry leaf diseases. However, despite the remarkable progress of these CNN-based models, their inherently limited local receptive fields hinder the modeling of global contextual information, leading to performance bottlenecks in complex backgrounds or when detecting weakly characterized lesions.

The development of plant disease detection has also been significantly driven by the availability of benchmark datasets. **Table 1** summarizes representative plant disease datasets in recent years. PlantVillage (Hughes and Salathé, 2015) is one of the earliest and most widely used datasets, containing 54,306 images across 38 disease categories from 14 crop species, primarily collected under controlled laboratory conditions. PlantDoc (Singh et al., 2020) addresses the limitation of laboratory-only images by providing 2,598 images captured in real-world field conditions, covering 27 disease classes across 13 plant species. For specific crops, datasets such as the Rice Disease Dataset (Lu et al., 2017) and Tomato Leaf Disease Dataset (Agarwal et al., 2020) have been developed to support targeted research. The IP102 dataset (Wu et al., 2019) focuses on insect pest recognition with 75,222 images covering 102 pest categories. However, most existing datasets are designed for image classification tasks rather than object detection, and few specifically target rubber tree diseases. To address this gap, we previously constructed PM-Dataset (Li et al., 2024) with 6,200 images for rubber tree powdery mildew detection. In this work, we further expand it to PM-Dataset-Plus with 9,765 images, and introduce PD-40 (Li et al., 2025), a large-scale multi-crop disease detection dataset with 80,369 images covering 40 disease categories across 8 crops, to comprehensively evaluate model performance and generalization capability.

**Table 1:** Summary of representative plant disease datasets.

Dataset	Images	Categories	Crops	Task	Environment
PlantVillage (Hughes and Salathé, 2015)	54,306	38	14	Cls.	Lab.
PlantDoc (Singh et al., 2020)	2,598	27	13	Cls.	Field
IP102 (Wu et al., 2019)	75,222	102	Multi.	Cls.	Mixed
Rice Disease (Lu et al., 2017)	5,932	10	1	Cls.	Field
Tomato Leaf (Agarwal et al., 2020)	18,160	10	1	Cls.	Lab.
PM-Dataset (Li et al., 2024)	6,200	6	1	Det.	Field
PM-Dataset-Plus	9,765	6	1	Det.	Field+Lab
PD-40 (Li et al., 2025)	80,369	40	8	Det.	Field

To overcome these limitations, the Transformer architecture has been widely adopted in the field of computer vision in recent years, demonstrating robust modeling capabilities in object detection tasks. The Vision Transformer (ViT) (Dosovitskiy et al., 2021) first demonstrated that a pure Transformer architecture can achieve competitive performance on image classification tasks when pre-trained on large-scale datasets. Subsequently, Swin Transformer (Liu et al., 2021) introduced hierarchical feature representation with shifted windows, achieving state-of-the-art results on various vision tasks including object detection. Since the introduction of the DETR model (Carion et al., 2020), a series of improved methods-including Deformable DETR (Zhu et al., 2020), DN-DETR (Li et al., 2022), and DINO (Zhang et al., 2022)-have been proposed successively. These models have achieved notable advancements in convergence speed, small-object detection, and feature representation capability. Among these methods, RT-DETR (Zhao et al., 2024) has emerged as a representative end-to-end detection framework that balances real-time performance and accuracy. By streamlining the decoder structure and integrating global-local feature perception, it effectively improves detection speed and generalization capability, making it a prominent example of Transformer-based object detection in recent years.

However, directly applying the Transformer architecture to plant disease detection still presents several challenges. On the one hand, lesion targets are typically small in size and variable in morphology, and standard Transformers remain limited in modeling small objects and fine-grained features. On the other hand, many Transformer-based models incur high computational overhead, which hinders their deployment on edge devices (Dananjayan et al., 2022). In addition, plant disease detection imposes higher demands on the model’s capabilities for multiscale feature fusion, local detail representation, and environmental adaptability.

To address the above challenges, this paper proposes RubberFormer, an efficient Transformer-based framework for detecting rubber tree powdery mildew, built upon an improved RT-DETR architecture. The proposed method integrates a lightweight backbone network, a multilevel attention fusion mechanism, and a cross-dimensional interaction module, with a focus on enhancing the detection of small lesions. By improving robustness and detection accuracy under complex backgrounds, RubberFormer aims to provide a more accurate, efficient, and deployable solution for disease monitoring in real-world agricultural environments.

## 3 Materials and methods

### 3.1 Data acquisition and processing

To address the limitations of the original PM-Dataset in representing diverse field conditions, we present PM-Dataset-Plus, an enhanced high-quality image dataset for detecting powdery mildew on rubber tree leaves. This new version expands our original 6,200 images (Li et al., 2024) to 9,765 carefully curated highresolution images, capturing a wider range of lesion appearances across different lighting, backgrounds, and scale variations, thereby enriching scene diversity and generalization capability for detection models.

**Figure 1:**
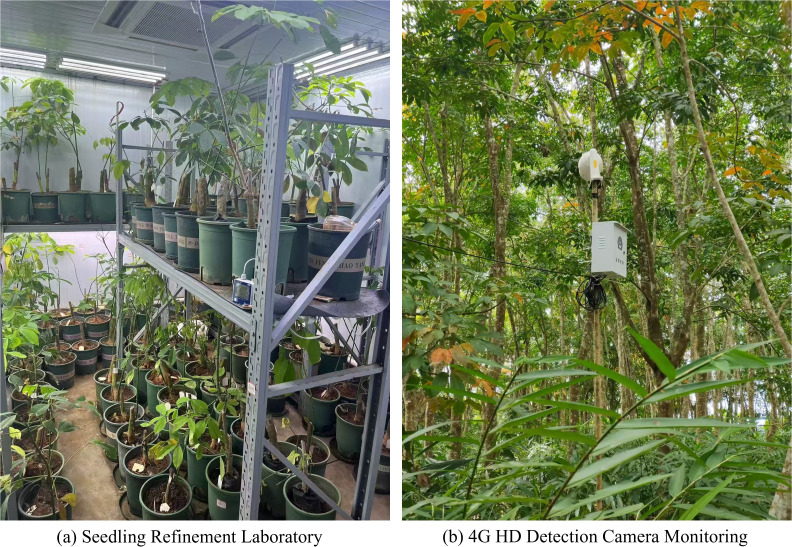
Data collection environment. **(a)** Controlled condition setup at the SRI laboratory, Hainan University. **(b)** 4G-enabled remote camera deployment at Jinjiang Farm.

Images were acquired from two distinct settings to ensure data diversity: natural field conditions across three major rubber-producing regions in Hainan (Baoting, Qiongzhong, and Danzhou), and a controlled laboratory environment at the Sanya Research Institute of Southern Propagation (SRI), Hainan University. The laboratory employed intelligent temperature and humidity controls to simulate diseaseprone environments, ensuring accurate depiction of powdery mildew lesions under both natural and controlled conditions, as shown in **Figure 1a**.

The image acquisition devices included high-resolution DSLR cameras such as Canon 6D Mark II and Nikon D90, as well as widely used mobile devices such as the iPhone 13, with image resolutions ranging from 4640×6960, 6240×4160, to 3120×3120, ensuring rich visual detail. Additionally, high-definition cameras with 4G communication capabilities (as shown in **Figure 1b**) were deployed at Jinjiang Farm in Baoting Li and Miao Autonomous County to enable remote visual monitoring and automated data collection. This setup not only supports scheduled acquisition and remote transmission of disease images but also provides key data for subsequent time-series analysis and early disease warning.

**Figure 2:**
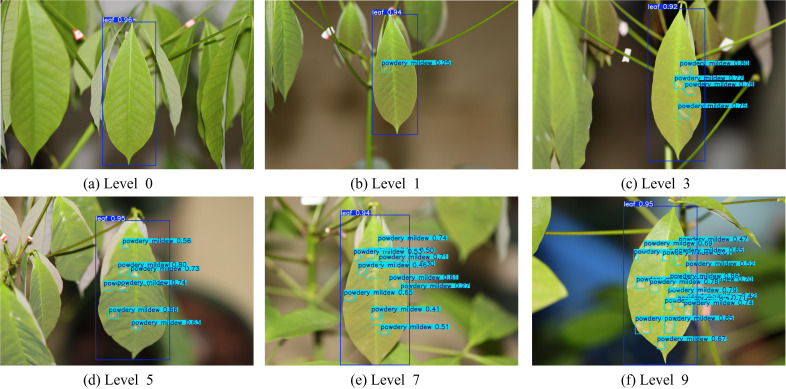
Representative samples of different disease severity levels in PM-Dataset-Plus. **(a)** Level 0: healthy leaf. **(b)** Level 1: initial infection. **(c)** Level 3: mild infection. **(d)** Level 5: moderate infection. **(e)** Level 7: severe infection. **(f)** Level 9: extremely severe infection.

After collection, the data were uniformly pre-processed through image quality screening, duplicate removal, blur detection, and lighting normalization. Each image was manually annotated using the LabelImg tool, with labels assigned to six disease severity levels: 0, 1, 3, 5, 7, and 9. This grading scheme follows the Chinese national standard for rubber tree powdery mildew investigation (GB/T 17980) (Standardization Administration of China, 2000), which defines disease severity based on the percentage of lesion coverage on individual leaves: Level 0 (no visible lesion), Level 1 (*<*5% coverage), Level 3 (5–15%), Level 5 (15–30%), Level 7 (30–50%), and Level 9 (*>*50%). The annotation process was carried out in collaboration with two agronomists from the School of Tropical Agriculture and Forestry, Hainan University, who provided expert guidance on boundary cases (e.g., distinguishing Level 0 healthy leaves from Level 1 initial infections with subtle lesions) and verified a random subset of 1,500 images (approximately 15%) to ensure annotation consistency, achieving an inter-annotator agreement (Cohen’s *κ*) of 0.87. **Figure 2** presents representative samples from each severity level, illustrating the visual characteristics of powdery mildew progression from healthy leaves (Level 0) to severely infected leaves (Level 9).

**Figure 3:**
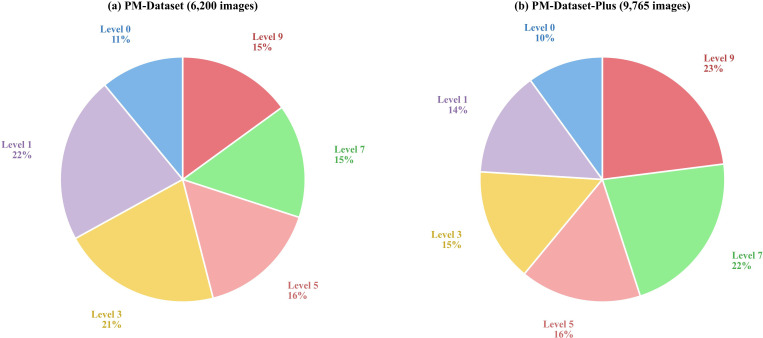
Distribution comparison between PM-Dataset and PM-Dataset-Plus. **(a)** PM-Dataset contains 6,200 images with a higher proportion of early-stage samples. **(b)** PM-Dataset-Plus expands to 9,765 images with increased severe disease samples to improve small object detection performance.

As illustrated in **Figure 3**, the original PM-Dataset exhibits a higher proportion of early-stage disease samples (Level 1: 22%, Level 3: 21%), while the expanded PM-Dataset-Plus significantly increases the proportion of severe disease samples (Level 7: 22%, Level 9: 23%). This augmentation strategy is specifically designed to enhance the model’s capability for detecting small and irregular lesions, as higher severity levels typically correspond to smaller and more fragmented lesion patterns that pose greater challenges for accurate detection.

The detailed distribution of severity levels is summarized in **Table 2**. In the original PM-Dataset, earlystage samples (Level 1 and Level 3) account for 43% of the total, while severe samples (Level 7 and Level 9) only comprise 30%. In contrast, PM-Dataset-Plus increases the proportion of severe samples to 45%, with Level 7 and Level 9 reaching 22% and 23%, respectively. This rebalancing strategy is motivated by the observation that higher severity levels typically exhibit smaller and more fragmented lesion patterns, which are more challenging for detection models. By augmenting these categories, we aim to improve the model’s robustness in small object detection scenarios.

**Table 2:** Comparison of data distribution between PM-Dataset and PM-Dataset-Plus.

Severity level	PM-Dataset		PM-Dataset-Plus	
	Count	Percentage	Count	Percentage
0	682	11.0%	977	10.0%
1	1,364	22.0%	1,367	14.0%
3	1,302	21.0%	1,465	15.0%
5	992	16.0%	1,563	16.0%
7	930	15.0%	2,149	22.0%
9	930	15.0%	2,244	23.0%
Total	6,200	100%	9,765	100%

**Figure 4:**
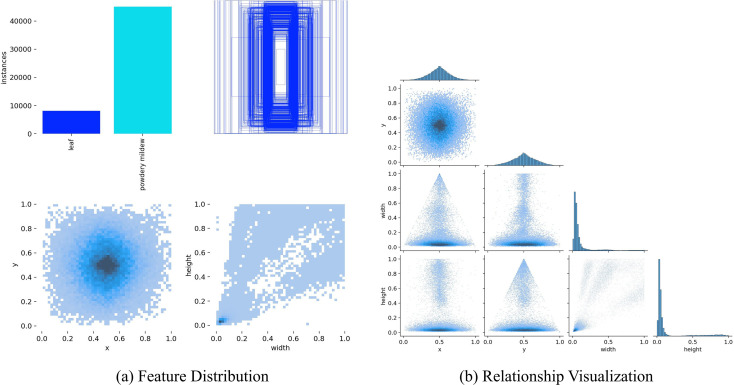
Statistical analysis of PM-Dataset-Plus annotations. **(a)** Feature Distribution: instance count per category, bounding box spatial distribution, center position heatmap, and width-height distribution heatmap. **(b)** Relationship Visualization: correlation matrix of bounding box attributes (x, y, width, height) with diagonal histograms and scatter plots showing inter-attribute relationships.

**Figure 4** presents a comprehensive statistical analysis of the annotation characteristics in PM-Dataset-Plus. As shown in **Figure 4(a)**, the feature distribution panel displays four key visualizations: the instance count per category (top-left), showing approximately 7,000 leaf instances and over 45,000 powdery mildew instances, reflecting the fine-grained nature of lesion annotation; the spatial distribution of all bounding boxes (top-right), revealing dense and overlapping annotations that correspond to the clustered appearance of powdery mildew lesions on leaf surfaces; the center point distribution heatmap (bottom-left), indicating that most lesions are concentrated in the central region of images with the highest density around coordinates (0.4, 0.5); and the width-height distribution heatmap (bottom-right), where a significant concentration of small-sized annotations is observed in the lower-left corner. **Figure 4(b)** presents a relationship visualization through a correlation matrix showing the relationships among bounding box attributes (x, y, width, height). The diagonal histograms reveal that both x and y coordinates follow approximately normal distributions centered around 0.5, while width and height distributions are heavily skewed toward smaller values, with most annotations having width and height below 0.2. This comprehensive analysis confirms the prevalence of small object detection challenges in this dataset, further justifying the design of our detection framework with enhanced small object perception capabilities.

To further evaluate the model’s generalization capability and cross-crop adaptability, we conducted comparative experiments on the PD-40 dataset (Li et al., 2025). PD-40 is a large-scale, diverse plant disease object detection dataset constructed to advance research in precision agriculture. It comprises 80,369 high-quality images with bounding box annotations, covering 40 disease categories across 8 major crops, including strawberry, tomato, peanut, rice, potato, rubber tree, wheat, and corn. It serves as a valuable complement to PM-Dataset-Plus due to its broad category coverage and high-quality annotations, and is used in this study to assess the generalization and robustness of the proposed detection framework.

### 3.2 RubberFormer

#### 3.2.1 RubberFormer network structure overview

**Figure 5:**
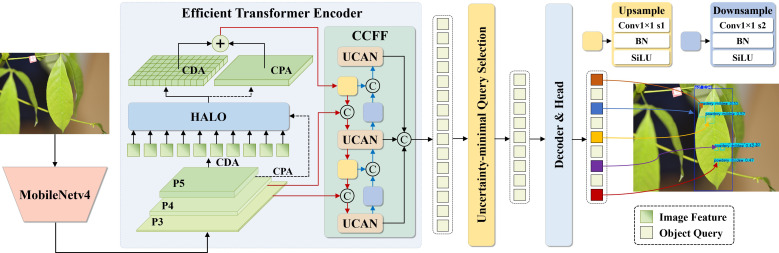
Overall architecture of the proposed RubberFormer network. The model integrates a lightweight MobileNetV4 backbone, a HALO-enhanced Transformer encoder, a UCAN module for spatial-channel interaction, and an uncertainty-based query selection mechanism.

In this study, we designed the RubberFormer network to enable end-to-end and accurate detection of rubber tree powdery mildew. The overall architecture is illustrated in **Figure 5**. The network adopts the highly efficient MobileNetV4 as its backbone, ensuring strong feature extraction capability while significantly reducing computational overhead, thus meeting the deployment requirements of edge devices.

Building on this foundation, we design an efficient Transformer encoder that integrates two key components: HALO, which performs adaptive fusion of multiscale features, and UCAN, which enhances the interaction between spatial and channel information. These two modules work in synergy to substantially improve the model’s ability to perceive and recognize tiny, irregular lesions under complex background conditions.

Finally, a query selection mechanism based on uncertainty minimization is applied to filter high-value information, and the decoder accurately outputs the detection results, forming a complete and efficient automated detection pipeline from input to prediction.

#### 3.2.2 MobileNetV4

MobileNetV4 synergizes the benefits of neural network architecture design and automated search methodologies to further augment the efficacy of vision tasks on mobile and edge devices. It introduces two key innovations in lightweight network design: the Universal Inverted Bottleneck (UIB) and the Mobile Multi-Query Attention (Mobile MQA), both of which significantly improve feature representation capability and hardware execution efficiency.

The UIB module is an evolution of the classical Inverted Bottleneck structure. By incorporating two searchable depthwise convolution operations, it generalizes several mainstream microstructures, such as the inverted residual block in MobileNetV2 (Sandler et al., 2018), the ConvNeXt block (Woo et al., 2023), and the feed-forward network (FFN) in Transformers.

Specifically, the computation within a UIB module involves the following steps: the input features are first expanded using a 1×1 convolution to increase the channel dimensionality; they are then optionally processed with a depthwise convolution for spatial modeling; finally, a 1×1 convolution is applied to compress the channel dimensions and produce the output. The generalized computational formulation is shown in Equation 1:

(1) $$X_{\text{out}}={\text{PWConv}}_{2}({\text{DWConv}}_{2}(\text{Act}({\text{DWConv}}_{1}({\text{PWConv}}_{1}(X)))))$$

where DWConv_1_ and DWConv_2_ are optional depthwise convolution operations whose activation is automatically determined by the neural architecture search (NAS) process. This mechanism allows the network to flexibly adjust its receptive field and computational cost based on task requirements. With this structure, MobileNetV4 achieves greater architectural diversity and enhanced feature modeling capability while maintaining computational efficiency, making it well-suited for small-object detection in complex environments, as required in this study.

In addition, to further enhance the network’s ability to model global information without incurring significant computational overhead, MobileNetV4 introduces the Mobile MQA mechanism. This module is derived from the conventional Multi-Head Self-Attention (MHSA), but optimized for mobile devices by sharing the Key and Value representations across all attention heads. This design significantly reduces memory access and computation costs. In the specific implementation, given an input feature *X*, the attention calculation and Mobile MQA output are shown in Equations 2, 3:

(2) $${\text{Attention}}_{j}=\text{softmax}(\frac{(XW_{j}^{Q}){\left(SR(X)W^{K}\right)}^{T}}{\sqrt{d_{k}}})(\text{SR}(X)W^{V})$$

(3) $$\text{Mobile}\_\text{MQA}(X)=\text{Concat}({\text{Attention}}_{1},\ldots ,{\text{Attention}}_{n})W^{O}$$

where SR(*X*) denotes the spatial reduction (downsampling) operation applied to the Key and Value features, formally defined as SR(*X*) = Reshape(*X,R*^2^*,C*) · *W^SR^*, where *R* is the spatial reduction ratio and $W^{SR}\in {\mathbb{R}}^{(C\cdot R^{2})\times C}$ is a learnable linear projection matrix. In practice, this is implemented as a 2 × 2 average pooling with stride 2 followed by a linear projection, which effectively reduces the spatial resolution of Key and Value tokens by a factor of 4, thereby reducing the computational complexity from *O*(*N*^2^) to *O*(*N*^2^*/R*^2^). This mechanism achieves substantial inference acceleration without significant performance impact and is particularly suitable for deploying and executing the attention mechanism on edge devices.

#### 3.2.3 HALO

**Figure 6:**
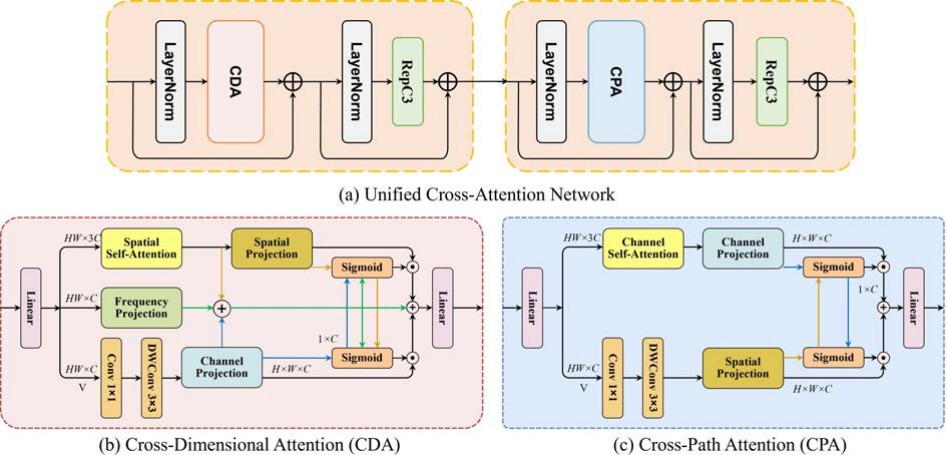
HALO Structure.

In object detection tasks, particularly in situations such as lesion detection where backgrounds are intricate and target sizes exhibit significant variability, enhancing the model’s feature extraction and robustness is crucial for reliable performance. In the specific context of rubber tree powdery mildew detection, lesion targets span a wide size range—from large leaf-level regions at early infection stages (Level 0–1) to tiny, scattered spore clusters at severe stages (Level 7–9)—requiring the model to simultaneously capture both fine-grained local textures and broader spatial context. Standard single-scale attention mechanisms struggle with this extreme scale variation. To this end, we propose the HALO module, as shown in **Figure 6**. This module performs hierarchical feature modeling and adaptive fusion through a Local-Global Attention mechanism. The local branch (*P* = 2) captures fine texture details critical for distinguishing subtle earlystage lesions from natural leaf patterns, while the global branch (*P* = 4) aggregates broader contextual information necessary for identifying large, contiguous infected regions. Together, they effectively enhance the ability of the model to represent small objects and boundary information and serve as a key feature enhancement component within the overall detection framework.

HALO module consists of three parallel branches: the local branch (Local, *P* = 2), the global branch (Global, *P* = 4), and the serial convolution branch. The input feature map $F\in {\mathbb{R}}^{H\times W\times C}$ is first channel-adjusted to obtain $F^{\prime }\in {\mathbb{R}}^{H\times W\times C^{\prime }}$, and then fed into each of the three branches to extract multiscale information. The local and global branches encode information within different receptive fields using Local-Global Attention. The patch token is created through non-overlapping window segmentation, followed by 302 local or global attention modeling achieved by channel averaging pooling, a feed-forward network, and 303 attention weighting. The attention weighting formula is shown in Equation 4:

(4) $${\tilde{t}}_{i}=P\cdot \text{sin }(\langle t_{i},\xi \rangle )\cdot t_{i}$$

where $t_{i}\in {\mathbb{R}}^{d}$ is the *i*-th token, $\xi \in {\mathbb{R}}^{C^{\prime }}$ is the task-related embedding vector, $P\in {\mathbb{R}}^{C^{\prime }\times C^{\prime }}$ is the learnable mapping matrix, and sin(·,·) denotes the normalized cosine similarity, which is used to measure the relevance between the current token and the task goal.

In addition, Local-Global Attention introduces a fusion mechanism of channel attention and spatial attention, inspired by the Convolutional Block Attention Module (CBAM) (Woo et al., 2018), to further enhance the modeling ability of key features, which is calculated as shown in Equation 5:

(5) $$F_{c}=M_{c}(\bar{F})⊗\bar{F},\;F_{s}=M_{s}(F_{c})⊗F_{c}$$

where $M_{c}$ and $M_{s}$ denote the channel attention map and spatial attention map, respectively, and ⊗ denotes element-by-element multiplication. $\bar{F}$ denotes the normalized or averaged value of the input feature F in thechannel dimension, which is used to enhance the stability and context-awareness of the attention module, and the formula is shown in Equation 6:

(6) $$\bar{F}={\text{Mean}}_{\text{Dim}=1}(F)$$

Meanwhile, the serial convolution branch simulates the spatial structure at different scales using multiple consecutive 3 × 3 convolution modules to add local details that the attention module might miss. The final output features of the three branches are *F*_local_, *F*_global_, and *F*_conv_, which are spliced in the channel dimension and then further refined by the convolution module containing the RepConv structure to output the final feature representation $F^{\prime \prime }\in {\mathbb{R}}^{H\times W\times C}$.

This module effectively integrates the multiscale features in the local and global receptive fields, providing robust and informative feature representations for subsequent tasks.

#### 3.2.4 UCAN

**Figure 7:**
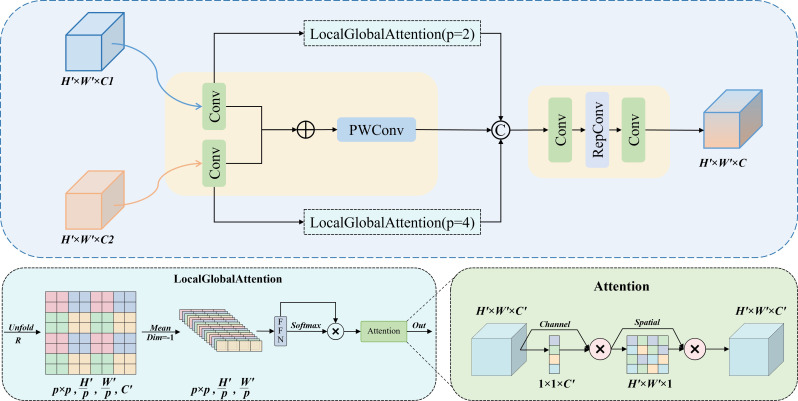
UCAN structure.

In this study, we propose UCAN, as shown in **Figure 7**, to address the insufficient interaction between different feature dimensions in the task of powdery mildew detection. Powdery mildew lesions exhibit subtle texture differences from healthy tissue—characterized by faint whitish powdery coatings and delicate vein discoloration—that require joint analysis across spatial locations, channel semantics, and frequency components to distinguish from visually similar artifacts such as water droplets, dust, or natural leaf variegation. This module enables collaborative modeling of spatial, channel, and frequency information by sequentially connecting two subcomponents: the Cross-Dimensional Attention (CDA) module and the Cross-Path Attention (CPA) module. The CDA module explicitly captures high-frequency edge features of lesion boundaries through its frequency path, while the CPA module models long-range channel dependencies to reinforce disease-relevant feature channels. Together, these components enhance the model’s ability to capture complex texture structures and local details that are essential for accurate powdery mildew identification.

Specifically, the CDA module first divides the input features $X\in {\mathbb{R}}^{H\times W\times C}$ into localized windows and performs self-attention operations in each window to compute the spatial response features. Subsequently, three types of auxiliary features—namely, spatial, channel, and frequency—are introduced to enhance the local perception capability and high-frequency information modeling through cross-dimensional weighted fusion.

Among them, the self-attention within each window in the CDA module is calculated by Equation 7:

(7) $${\text{Y}}_{\text{DA}}^{\left(i\right)}=\text{Softmax}\left(\frac{\left({\text{Q}}^{\left(i\right)}{\text{K}}^{{\left(i\right)}^{⊤}}\right)}{\sqrt{d}}+\text{B}\right){\text{V}}^{\left(i\right)}$$

Spatial, channel, and frequency mapping features are defined in Equation 8:

(8) $${\text{Y}}_{S}=f_{\text{SP}}({\text{Y}}_{\text{DA}}),\;{\text{Y}}_{C}=f_{\text{CP}}(X),\;{\text{Y}}_{F}=f_{\text{FP}}(\text{Linear}(X))$$

The final output of the CDA module is shown in Equation 9:

(9) $${\text{Y}}_{\text{CDA}}={\text{Y}}_{\text{DA}}\cdot \sigma (\text{AvgPool}({\text{Y}}_{F}\cdot {\text{Y}}_{C}))+{\text{Y}}_{C}\cdot \sigma ({\text{Y}}_{F}\cdot {\text{Y}}_{S})+{\text{Y}}_{F}$$

where *σ*(·) denotes the Sigmoid function, and AvgPool is used to extract the joint channel-frequency statistical information and generate the enhanced expression after fusing the multidimensional information.

To further model the long-range dependencies along the channel dimension, the CPA module applies a self-attention mechanism to the channel-expanded representation of the input features, thereby obtaining the backbone channel response. Additionally, a spatial modulation branch and a local convolutional enhancement path (Dai et al., 2024) are introduced to integrate channel modeling with spatial structure perception. The channel attention in the CPA module is computed as shown in Equation 10:

(10) $${\text{Y}}_{\text{PA}}=\text{Softmax}(\frac{{\text{Q}}_{C}^{⊤}{\text{K}}_{C}}{\alpha }){\text{V}}_{C}$$

where $Q_{C},{\text{K}}_{\text{C}},{\text{V}}_{C}\in {\mathbb{R}}^{C\times (H\cdot W)}$ are learnable temperature-scaled parameters, and the output **Y**_PA_ is reshaped back to ${\mathbb{R}}^{H\times W\times C}$. The spatial modulation and channel enhancement features are defined in Equation 11:

(11) $${\text{Y}}_{S}=f_{\text{SP}}(X),\;{\text{Y}}_{C1}=f_{\text{C}1}({\text{Y}}_{\text{PA}}),\;{\text{Y}}_{C2}=f_{\text{C}2}({\text{Y}}_{\text{PA}})$$

The final output of the CPA module is denoted in Equation 12:

(12) $${\text{Y}}_{\text{CPA}}={\text{Y}}_{\text{PA}}+{\text{Y}}_{C1}\cdot \sigma ({\text{Y}}_{S})+\text{DWConv}({\text{V}}_{C})\cdot \sigma ({\text{Y}}_{C2})$$

where DWConv denotes the depth separable convolution operation, and dot product is element-by-element multiplication. Through the cross-fertilization of the main channel attention with the two auxiliary paths, the CPA module achieves unified modeling of channel semantics, spatial structure and local details.

By connecting the CDA and CPA modules in series, UCAN realizes the deep fusion of spatial, channel and frequency information in the feature extraction backbone, which significantly improves the model’s ability to respond to complex lesions and the recognition accuracy, and effectively alleviates the bias problem of the traditional attention mechanism on low-frequency structures.

#### 3.2.5 Normalized Wasserstein distance loss

In the task of detecting rubber tree powdery mildew, lesions typically exhibit small size, irregular shape, and fuzzy boundaries, resulting in the lesion region occupying only a small number of pixels within the image. As shown in our dataset analysis, over 74% of bounding boxes at severe infection levels (Level 7–9) qualify as small objects (area *<* 32^2^ pixels), and even a minor localization offset of a few pixels can drastically alter the IoU value. Traditional IoU-based metrics and their extensions (e.g., GIoU, DIoU, CIoU) have long been used to measure the similarity between predicted and ground-truth bounding boxes. However, these metrics are highly sensitive to localization errors in small-object detection, often leading to significant performance degradation when lesions are tiny and imprecisely located. While Focal Loss (Lin et al., 2017b) addresses class imbalance by down-weighting easy examples, it does not directly solve the localization sensitivity problem for small objects.

To alleviate this problem, NWD Loss is introduced as a more robust and stable alternative for bounding box regression. The core idea is to model the target boxes as two-dimensional Gaussian distributions and use the Wasserstein distance between the Gaussian distributions to measure their similarity. Specifically, for any two bounding boxes *A* = (*c_x_,c_y_,w_a_,h_a_*) and *B* = (*c_x_,c_y_,w_b_,h_b_*), they are modeled as two-dimensional Gaussian distributions N*_a_*and N*_b_*, respectively, where the *centroid* is the center of the bounding box, and the width and height determine the covariance matrix in the form shown in Equation 13:

(13) $$\mu =[\begin{array}{c} c_{x}\\ c_{y} \end{array}],\text{ Σ}=[\begin{array}{cc} {\left(w/2\right)}^{2} & 0\\ 0 & {\left(h/2\right)}^{2} \end{array}]$$

The Wasserstein distance between the two is shown in Equation 14:

(14) $$W_{2}^{2}\left(N_{a},N_{b}\right)={\left\|\mu_{a}-\mu_{b}\right\|}_{2}^{2}+\;{\left\|{\text{Σ}}_{a}^{1/2}-{\text{Σ}}_{b}^{1/2}\right\|}_{F}^{2}$$

In practical applications such as powdery mildew small-target detection, in order to make this distance more suitable as a loss function (and ensure the result lies in [0,1]), a normalized exponential function is applied to obtain the NWD Loss, as shown in Equation 15:

(15) $$\text{NWD}(N_{a},N_{b})=\text{exp }(-\frac{\sqrt{W_{2}^{2}(N_{a},N_{b})}}{C})$$

where the constant *C* is a normalization factor related to the dataset scale. In this study, *C* is set to the average diagonal length of all ground-truth bounding boxes in the training set: *C* = 12.8 pixels for PM-Dataset-Plus and *C* = 18.5 pixels for PD-40, reflecting the different average target sizes across the two datasets. To evaluate sensitivity to this hyperparameter, we performed an ablation over *C* ∈ {8,10,12.8,16,20} on PM-Dataset-Plus; the mAP_50_ varied within a narrow range of 87.8–88.3%, indicating that the model is robust to the choice of *C* within a reasonable range around the dataset-specific average. NWD is incorporated into the regression loss, the positive/negative sample assignment strategy, and the non-maximum suppression step. Compared to IoU, NWD Loss not only provides valid similarity even when boxes do not overlap but also introduces a fine-grained distance representation, which benefits optimization stability and convergence.

## 4 Experimental setup and results

### 4.1 Experiment configuration

All experiments in this study were conducted on a server cluster equipped with eight NVIDIA GeForce RTX 3090 GPUs, each with 24 GB of memory. The system runs Ubuntu 22.04, and all models were implemented and trained using the PyTorch framework in Python. The RubberFormer model was trained on a self-constructed rubber tree powdery mildew dataset called PM-Dataset-Plus, which was split into training, validation, and test sets in an 8:1:1 ratio using a stratified sampling strategy to preserve the proportion of each severity level (0, 1, 3, 5, 7, 9) across all three subsets, thereby avoiding evaluation bias caused by class imbalance. The training process employed the AdamW optimizer with an initial learning rate of 0.0001, momentum set to 0.9, and a weight decay of 0.0001. The model was trained for 500 epochs with a batch size of 32. Pretrained weights were loaded to accelerate convergence, and the random seed was set to 0 to ensure deterministic behavior and reproducibility.

To balance efficiency and performance, mixed-precision training (AMP) and mosaic augmentation were disabled. Data augmentation was applied online during training with the following settings: random horizontal flipping (probability 0.5), random scaling (scale factor uniformly sampled from [0.8, 1.2]), and random translation (shift ratio up to ±10% of image width and height). No offline augmentation was performed; therefore, the effective training set size remains 7,812 images per epoch (80% of 9,765), with augmentation applied stochastically to each sample during loading. During validation, non-maximum suppression (NMS) with an IoU threshold of 0.7 was enabled. Throughout training, checkpoint saving, metric visualization, and logging were activated to facilitate model debugging and performance comparison. The final trained model was exported in TorchScript format for optimized inference and deployment on edge devices.

### 4.2 Evaluation metrics

To comprehensively evaluate the proposed RubberFormer model, we employ a set of accuracy metrics including mean Average Precision (mAP) under various IoU thresholds, Precision, Recall, and F1-score. In addition, computational indicators such as Frames Per Second (FPS), Floating Point Operations (FLOPs), and parameter count (Param) are used to assess efficiency. FPS is measured as the reciprocal of the average per-image inference time (i.e., FPS = 1*/t*_inference_), where *t*_inference_ includes pre-processing, forward pass, and post-processing (NMS). All FPS values reported in this study were measured on a single NVIDIA GeForce RTX 3090 GPU with a batch size of 1, averaged over the entire test set after a 100-image warm-up to exclude initialization overhead. These metrics together provide a holistic view of the model’s accuracy and computational performance. The specific definitions and calculation methods are presented in Equations 16–20:

(16) $$\text{Precision}=\frac{TP}{TP+FP},\text{ Recall}=\frac{TP}{TP+FN}$$

(17) $$\text{F}1-\text{score}=2\cdot \frac{P\cdot R}{P+R}$$

(18) $$\text{AP}=\int_{0}^{1}P(r)\;dr$$

(19) $${\text{mAP}}_{50}=\frac{1}{N}\sum_{i=1}^{N}\underset{\text{IoU}\in \{0.5\}}{\max}{\text{AP}}_{i}$$

(20) $${\text{mAP}}_{50-95}=\frac{1}{N}\sum_{i=1}^{N}\underset{\text{IoU}\in \{0.5:0.05:0.95\}}{\max}{\text{AP}}_{i}$$

where: *TP* is the number of true positive samples; *FP* is the number of false positive samples; *FN* is the number of false negative samples; *P* is Precision, *R* is Recall; *P*(*r*) is the precision as a function of Recall *r*; *N* is the total number of classes; and AP*_i_* is the Average Precision for class *i*.

### 4.3 Detection results of RubberFormer

#### 4.3.1 Comparative experiment on PM-Dataset-Plus

In the comparative experiment of this study, we systematically assess the performance of several mainstream target detection methods, including Faster R-CNN, SSD, YOLO series, DINO, Deformable DETR, and RT-DETR, on the PM-Dataset-Plus dataset. The experimental results are shown in **Table 3**. Overall, RubberFormer achieved the best performance across multiple evaluation metrics: mAP_50_ of 88.3%, mAP_50–95_ of 65.1%, Precision of 87.9%, Recall of 86.7%, and a high F1-score of 87.3%. Compared to the similarly strong RT-DETR-X (F1-score of 85.5%), RubberFormer significantly reduced computational overhead (76.6G FLOPs vs. 222.5G) and improved inference efficiency (50.8 FPS), while maintaining high accuracy. These results demonstrate that RubberFormer is better suited for real-world applications that demand both computational efficiency and high throughput, without compromising detection accuracy.

**Table 3:** Comparative experiment results on the PM-Dataset-Plus.

Method	mAP_50_(%)	mAP_50-95_(%)	Precision(%)	Recall(%)	F1(%)	FLOPs(G)	FPS
Two-stage Detector							
Faster-RCNN	79.8	60.5	78.6	76.8	77.7	201.7	35.5
One-stage CNN-based Detectors							
SSD	75.9	58.2	75.1	71.7	73.4	30.5	79.6
YOLOv5	81.9	61.4	82.2	77.0	79.5	7.2	87.3
YOLOv7	81.9	62.3	84.1	79.8	81.9	103.8	87.7
YOLOv8	82.9	62.6	84.4	80.2	82.2	8.2	88.7
YOLOv10	81.2	61.4	81.7	77.0	79.3	6.5	128.9
YOLOv11	81.0	61.8	82.8	77.3	79.9	6.3	119.2
YOLOv12	83.1	62.3	84.0	79.2	81.5	5.8	70.0
Transformer-based Detectors							
DINO	80.9	63.0	83.6	80.5	82.0	231.7	29.8
Deformable DETR	83.5	64.8	84.5	81.8	83.2	184.0	31.1
RT-DETR-r18	81.5	61.2	83.3	77.3	80.2	56.9	39.7
RT-DETR-L	84.9	63.9	85.1	81.0	83.0	103.4	28.1
RT-DETR-r50	83.1	62.3	84.0	81.9	82.9	129.5	28.3
RT-DETR-r101	83.2	62.2	84.7	80.0	82.3	247.1	13.2
RT-DETR-X	85.1	64.5	87.1	84.0	85.5	222.5	16.0
Proposed Method							
**RubberFormer**	**88.3**	**65.1**	**87.9**	**86.7**	**87.3**	**76.6**	**50.8**

Best results are in bold.

Additionally, compared with classical methods such as Faster R-CNN and SSD, RubberFormer shows substantial improvements in accuracy, Recall, and F1-score, highlighting its superior feature extraction and object perception capabilities. When compared with the YOLO series, RubberFormer further improves detection accuracy while maintaining a lightweight architecture, and outperforms them on key metrics such as mAP_50_ and F1-score. Notably, RubberFormer also demonstrates higher accuracy and greater efficiency than high-precision Transformer-based models such as Deformable DETR and RT-DETR, validating the effectiveness and robustness of its architectural design.

**Figure 8:**
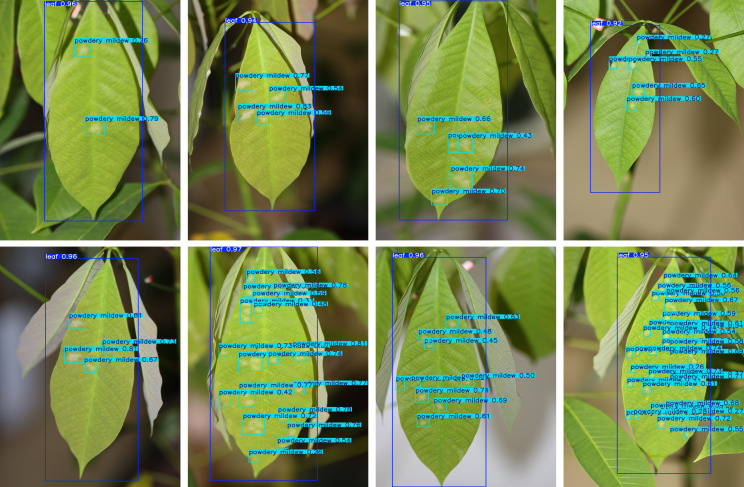
Detection results of different severity levels in PM-Dataset-Plus. **(a)** Level 0. **(b)** Level 1. **(c)** Level 3. **(d)** Level 5. **(e)** Level 7. **(f)** Level 9.

To further demonstrate the model’s performance, we performed a hierarchical visualization analysis of the leaf detection results for different disease classes, as shown in **Figure 8**, to verify the model’s reliability and robustness in terms of its ability to discriminate between different disease classes.

It is worth noting that PM-YOLO (Li et al., 2024), a method previously designed for the same task, reported 86.9% mAP on the original PM-Dataset. However, PM-YOLO was built upon YOLOv10 with task-specific feature fusion modifications and has not been re-implemented on PM-Dataset-Plus by the original authors. To provide an approximate reference, we trained YOLOv10 (the base architecture of PM-YOLO) on PM-Dataset-Plus, which achieved 81.2% mAP_50_. RubberFormer outperforms this baseline by 7.1%, suggesting a substantial improvement over the PM-YOLO lineage. A full re-implementation of PM-YOLO with its proprietary modules on PM-Dataset-Plus is left for future work pending code availability.

**Figure 9:**
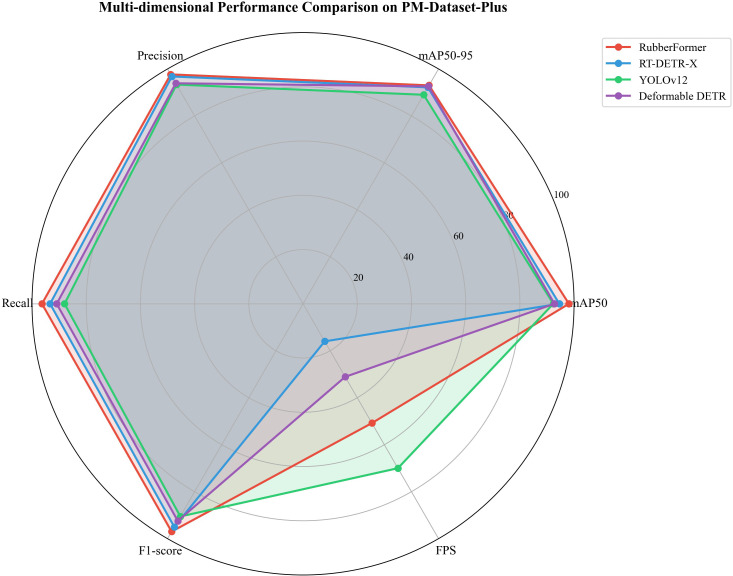
Multi-dimensional performance comparison of RubberFormer with representative detection methods on PM-Dataset-Plus. The radar chart visualizes six key metrics: mAP50, mAP50-95, Precision, Recall, F1-score, and FPS.

To comprehensively evaluate RubberFormer’s performance across multiple dimensions, **Figure 9** presents a radar chart comparing RubberFormer with representative methods including RT-DETR-X, YOLOv12, and Deformable DETR on PM-Dataset-Plus. The visualization demonstrates that RubberFormer achieves superior performance across all evaluation metrics, particularly excelling in mAP50, Recall, and F1-score while maintaining competitive inference speed.

**Figure 10:**
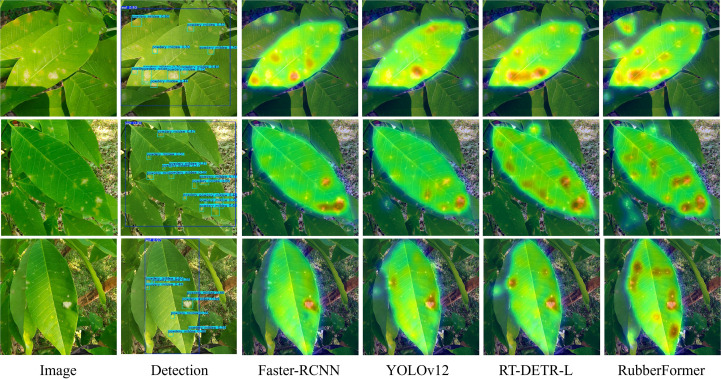
Detection results and Grad-CAM heatmap visualization for different models on PM-Dataset-Plus. From left to right: original image, detection result, and heatmaps of Faster-RCNN, YOLOv12, RT-DETR-L, and RubberFormer. RubberFormer shows stronger focus on lesion regions under varying backgrounds.

After visualizing the detection results of each method on PM-Dataset-Plus, we used Grad-CAM to visualize the heat map outputs of the four different models, as shown in **Figure 10**. The heatmaps demonstrate the degree of attention paid by the different models to the diseased spot area. As observed, RubberFormer can still accurately recognize most of the lesion area, showing stronger focus on the actual disease regions compared to other methods.

**Figure 11:**
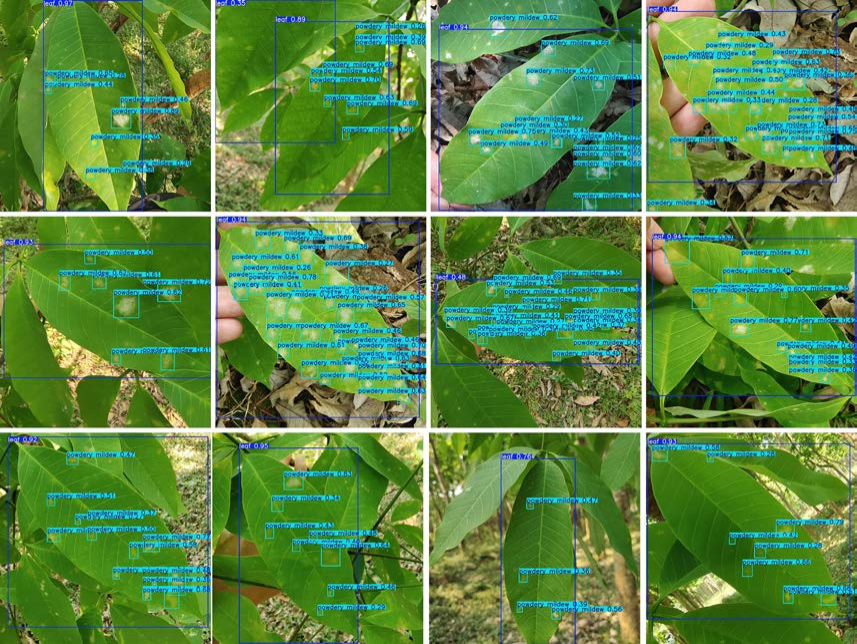
Detection results under complex environments and high-density lesion distributions in PMDataset-Plus, demonstrating RubberFormer’s robustness in challenging real-world scenarios.

**Figure 8** presents the detection results across different severity levels in PM-Dataset-Plus, demonstrating RubberFormer’s capability to accurately identify lesions ranging from mild (Level 0-3) to severe (Level 7-9) infections. Furthermore, **Figure 11** showcases the detection performance under challenging conditions, including complex backgrounds, high-density lesion distributions, and varying lighting conditions. These results confirm the robustness of RubberFormer in real-world agricultural scenarios.

In addition, we also conducted comparative experiments of the RubberFormer model on the PM-Dataset dataset separately to verify the effect of dataset expansion on model performance enhancement. The results are shown in **Table 4**. Compared with the original PM-Dataset, the detection performance of the model on PM-Dataset-Plus was significantly improved, and both mAP_50_ and F1-score were improved by 0.7%. This result indicates that the expanded dataset provides more comprehensive training support in terms of sample number, category distribution and scene diversity, which further enhances the model’s ability to generalize to complex backgrounds and disease variants.

**Table 4:** Performance comparison between PM-Dataset and PM-Dataset-Plus.

Dataset	Number	mAP50(%)	mAP50-95(%)	Precision(%)	Recall(%)	F1-score(%)
PM-Dataset	6200	87.6	64.8	87.4	86.0	86.6
PM-Dataset-Plus	9765	88.3	65.1	87.9	86.7	87.3

#### 4.3.2 Comparative experiment on PD-40 Dataset

To evaluate the generalization ability and robustness of RubberFormer under complex conditions, we conducted a comparative experiment on the PD-40 dataset (Li et al., 2025). As shown in **Table 5**, RubberFormer outperforms other methods across multiple evaluation metrics, demonstrating clear advantages. Specifically, it achieves 93.5% mAP_50_ and 80.2% mAP_50-95_, surpassing mainstream CNNbased detectors such as YOLOv12 and Transformer-based detectors such as RT-DETR-X, indicating stronger accuracy and stability in plant disease target recognition.

**Table 5:** Comparative experiment results on the PD-40 Dataset.

Method	mAP_50_(%)	mAP_50-95_(%)	Precision(%)	Recall(%)	F1(%)	FLOPs(G)	FPS
Two-stage Detector							
Faster-RCNN	82.1	65.3	83.2	79.5	81.3	201.7	32.4
One-stage CNN-based Detectors							
SSD	78.6	61.8	79.4	75.2	77.2	30.5	75.3
YOLOv5	85.4	68.7	86.1	82.3	84.2	7.2	82.6
YOLOv7	86.8	70.2	87.5	83.6	85.5	103.8	81.4
YOLOv8	87.9	71.5	88.3	84.8	86.5	8.2	83.2
YOLOv10	88.5	72.3	88.9	85.2	87.0	6.5	115.6
YOLOv11	89.2	73.1	89.5	85.8	87.6	6.3	108.3
YOLOv12	90.1	74.2	90.3	86.5	88.4	5.8	65.2
Transformer-based Detectors							
DINO	86.5	70.8	87.2	83.9	85.5	231.7	27.5
Deformable DETR	88.2	72.6	88.8	85.1	86.9	184.0	28.9
RT-DETR-r18	87.3	71.4	87.9	84.2	86.0	56.9	36.8
RT-DETR-L	89.6	73.8	90.1	86.3	88.2	103.4	26.4
RT-DETR-r50	88.9	72.9	89.4	85.7	87.5	129.5	26.1
RT-DETR-r101	89.1	73.2	89.6	85.9	87.7	247.1	12.5
RT-DETR-X	91.2	75.6	91.5	87.8	89.6	222.5	14.8
Proposed Method							
**RubberFormer**	**93.5**	**80.2**	**92.8**	**89.5**	**91.1**	**76.6**	**48.6**

Best results are in bold.

Moreover, RubberFormer’s balanced performance in Precision (92.8%) and Recall (89.5%) further confirms its detection robustness in high-complexity, multi-category scenarios. Although its inference speed of 48.6 FPS is not the fastest among all methods, its overall performance is exceptional, as it effectively balances detection accuracy and computational cost while maintaining a practical inference rate. These results further demonstrate that RubberFormer is robust across diverse scenarios, exhibits strong adaptability, and offers practical feasibility and advantages for real-world applications such as intelligent agricultural monitoring.

**Figure 12:**
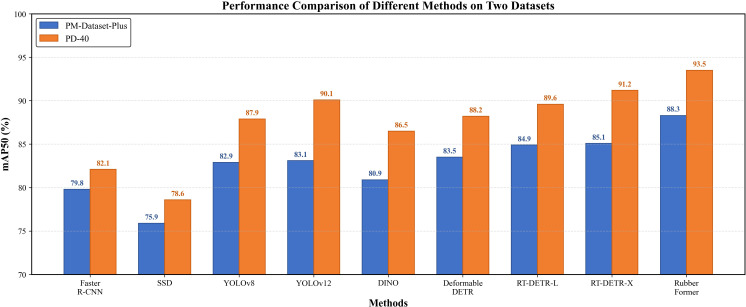
Performance comparison (mAP50) of different detection methods on PM-Dataset-Plus and PD-40 datasets. RubberFormer achieves the highest accuracy on both datasets.

**Figure 12** provides a comparative analysis of different methods on both PM-Dataset-Plus and PD-40 datasets. The grouped bar chart reveals that RubberFormer consistently outperforms all baseline methods on both datasets, achieving 88.3% mAP50 on PM-Dataset-Plus and 93.5% on PD-40. This consistent superiority across datasets with different characteristics demonstrates the strong generalization capability of the proposed framework.

**Figure 13:**
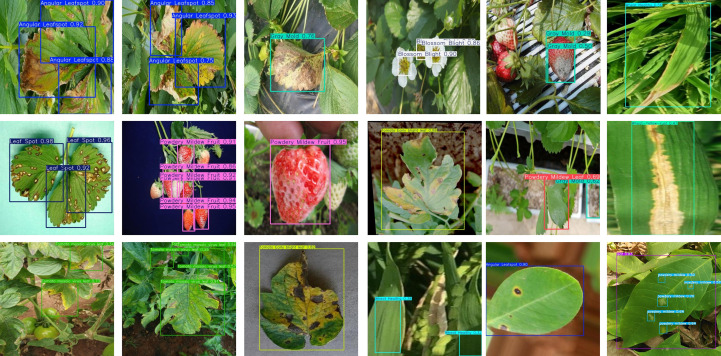
Detection results of RubberFormer on the PD-40 dataset, demonstrating accurate localization and classification across diverse plant disease categories.

**Figure 14:**
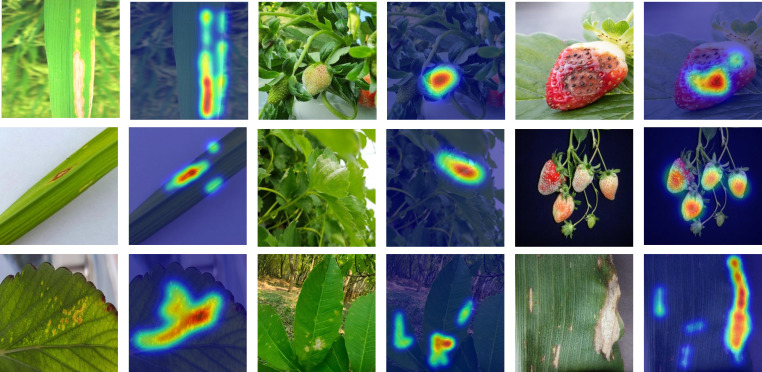
Grad-CAM heatmap visualization of RubberFormer on the PD-40 dataset, showing focused attention on disease regions across different crop species.

To further illustrate the detection performance on the PD-40 dataset, **Figure 13** presents representative detection results across various plant disease categories. The visualization demonstrates that RubberFormer can accurately localize and classify diverse disease types with high confidence scores. Additionally, **Figure 14** shows the Grad-CAM heatmap visualization on PD-40, where RubberFormer exhibits focused attention on the actual disease regions, confirming its strong feature extraction capability across different crop species.

#### 4.3.3 Ablation experiments

To further assess the specific contribution of each module within RubberFormer to the overall performance, we conducted systematic ablation experiments on the PM-Dataset-Plus, with the results summarized in **Table 6**. RT-DETR-L was used as the baseline and the MobileNetV4, HALO, UCAN, and NWD Loss modules were incrementally introduced to observe the impact of different combinations on detection accuracy and computational efficiency.

**Table 6:** Ablation experiment results on PM-Dataset-Plus.

Baseline	MobileNetV4	HALO	UCAN	NWD loss	mAP50 (%)	Precision (%)	Recall (%)	F1-score (%)	FPS	FLOPs	Param
✓					84.9	85.1	81.0	83.0	28.1	103.4G	3198.8M
✓	✓				84.1	84.0	80.7	82.3	70.8	40.9G	1161.6M
✓	✓	✓			85.8	85.9	81.2	83.5	58.6	69.6G	1823.9M
✓	✓	✓	✓		87.3	86.5	85.5	86.0	52.1	72.9G	2040.3M
✓	✓	✓	✓	✓	**88.3**	**87.9**	**86.7**	**87.3**	50.8	76.6G	2088.9M

Bold values indicate the best result in each column.

The experimental results show that introducing MobileNetV4 alone significantly reduces the model’s computational overhead, improving the FPS from 28.1 to 70.8. Both FLOPs and the number of parameters are also considerably reduced, demonstrating strong lightweight capability. With the addition of the HALO module, the mAP50 improves substantially to 85.8%, and the F1-score reaches 83.5%, indicating the module’s effectiveness in enhancing multiscale perception and feature modeling. When UCAN is further integrated, the mAP50 increases to 87.3% while only incurring a moderate increase in computational cost. This highlights the favorable balance UCAN achieves between performance and efficiency in modeling cross-dimensional interactions. Finally, when integrating NWD Loss for optimal small target detection, the model achieves the highest mAP50 (88.3%) with F1-score (87.3%), validating the robust localization capability of NWD Loss for small-scale targets.

Nevertheless, as modules are accumulated, the computational resource requirements of the model progressively increase, resulting in a reduction of the final FPS to 50.8. Nonetheless, it remains within a practical range for deployment. In summary, RubberFormer demonstrates a strong synergistic effect among its components, particularly in feature enhancement and small target modeling, which substantially elevates overall performance and confirms the rationality and efficacy of its design.

**Figure 15:**
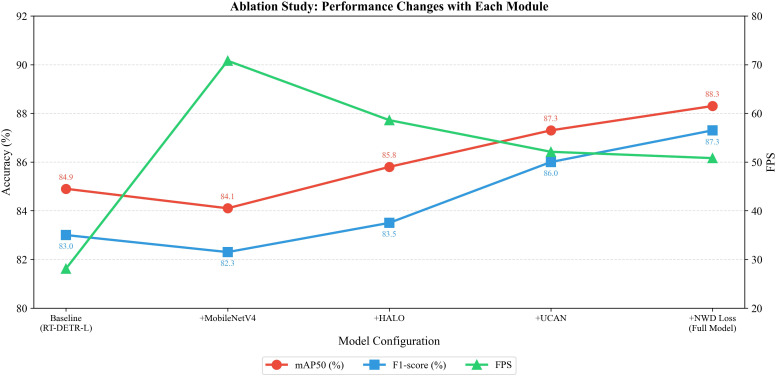
Ablation study visualization showing the performance changes (mAP50, F1-score, and FPS) as each module is progressively integrated into the RubberFormer framework.

To provide a more intuitive understanding of the ablation study results, **Figure 15** visualizes the performance changes as each module is progressively added. The line chart clearly shows that while MobileNetV4 significantly improves inference speed (FPS increases from 28.1 to 70.8), the subsequent addition of HALO, UCAN, and NWD Loss modules progressively enhances detection accuracy (mAP50 and F1-score) with only moderate reductions in speed, ultimately achieving an optimal balance in the full RubberFormer model.

#### 4.3.4 Analysis of detection performance across severity levels

While the overall performance metrics demonstrate RubberFormer’s superiority, understanding how the model performs across different disease severity levels is crucial for practical applications. In real-world scenarios, agricultural monitoring systems must reliably detect diseases at all stages of progression, from early-stage infections that are critical for timely intervention to severe cases that require immediate attention. To comprehensively evaluate this capability, we conducted a detailed per-class analysis on PM-Dataset-Plus. **Table 7** presents the detection performance metrics for each severity level (0, 1, 3, 5, 7, and 9), providing insights into the model’s behavior under varying disease conditions.

**Table 7:** Per-class detection performance of RubberFormer on PM-Dataset-Plus across different severity levels.

Severity level	AP_50_(%)	AP_50-95_(%)	Precision(%)	Recall(%)	F1(%)	Instances
Level 0 (Healthy)	95.2	78.6	94.1	93.8	93.9	977
Level 1 (Initial)	89.7	66.3	88.5	87.2	87.8	1,367
Level 3 (Mild)	88.1	64.8	87.3	86.5	86.9	1,465
Level 5 (Moderate)	87.5	63.9	86.8	85.9	86.3	1,563
Level 7 (Severe)	86.3	62.1	85.9	84.7	85.3	2,149
Level 9 (Extreme)	83.2	58.7	84.2	82.3	83.2	2,244
Average	88.3	65.1	87.9	86.7	87.3	9,765

The results reveal several important observations. The model achieves the highest detection accuracy on healthy leaves (Level 0), with an AP_50_ of 95.2%, which is expected as healthy leaves have distinct visual characteristics that are easier to distinguish. As the severity level increases from Level 1 to Level 9, the detection performance gradually decreases. This trend is particularly evident in the transition from Level 7 to Level 9, where the AP_50_ drops from 86.3% to 83.2%. To quantitatively support this observation, **Table 8** presents the average bounding box size statistics for each severity level.

**Table 8:** Average bounding box size statistics per severity level on PM-Dataset-Plus.

Severity level	Avg. width (px)	Avg. height (px)	Avg. area (px^2^)	Small object ratio (%)
Level 0 (Healthy)	186.3	201.5	37,539	8.2
Level 1 (Initial)	72.8	68.4	4,980	35.6
Level 3 (Mild)	58.5	54.2	3,171	48.3
Level 5 (Moderate)	45.7	42.8	1,956	61.7
Level 7 (Severe)	32.4	29.6	959	74.5
Level 9 (Extreme)	24.1	21.8	525	82.9

As shown in **Table 8**, the average bounding box area decreases dramatically from 37,539 px^2^ at Level 0 to only 525 px^2^ at Level 9, representing a 71.5× reduction. The small object ratio (defined as bounding boxes with area *<* 32^2^ pixels following the COCO small object convention) increases from 8.2% at Level 0 to 82.9% at Level 9. This quantitative evidence confirms that higher severity levels correspond to significantly smaller and more fragmented lesion patterns, directly explaining the observed performance decline.

Despite this challenge, RubberFormer maintains relatively stable performance across all severity levels, with the performance gap between the best (Level 0) and worst (Level 9) cases being only 12.0% in terms of AP_50_. This demonstrates the robustness of the proposed framework in handling diverse disease manifestations. The consistent performance across severity levels is largely attributed to the HALO module’s multiscale feature fusion capability and the NWD Loss’s effectiveness in handling small object localization. These results suggest that RubberFormer can serve as a reliable tool for comprehensive disease assessment across the entire spectrum of infection severity.

#### 4.3.5 Computational efficiency analysis

Beyond detection accuracy, computational efficiency is equally important for practical deployment in agricultural monitoring scenarios. Modern precision agriculture increasingly relies on edge computing devices such as agricultural drones, mobile monitoring stations, and embedded systems that have limited computational resources. Therefore, we conducted a comprehensive analysis of the computational requirements and inference efficiency of RubberFormer compared to other state-of-the-art methods. **Table 9** presents a detailed comparison of model complexity metrics, including parameter count, FLOPs, inference speed, GPU memory consumption, and latency.

**Table 9:** Computational efficiency comparison of different detection methods.

Method	Params (M)	FLOPs (G)	FPS	GPU memory (GB)	Latency (ms)	mAP_50_(%)	Efficiency score
Faster-RCNN	41.5	201.7	35.5	4.8	28.2	79.8	0.40
YOLOv8	11.2	8.2	88.7	2.1	11.3	82.9	10.11
YOLOv12	9.8	5.8	70.0	1.9	14.3	83.1	14.33
DINO	47.2	231.7	29.8	6.2	33.6	80.9	0.35
Deformable DETR	40.1	184.0	31.1	5.5	32.2	83.5	0.45
RT-DETR-L	32.0	103.4	28.1	4.1	35.6	84.9	0.82
RT-DETR-X	67.5	222.5	16.0	7.3	62.5	85.1	0.38
RubberFormer	20.9	76.6	50.8	3.2	19.7	**88.3**	1.15

Bold values indicate the best result in each column.

The Efficiency Score is calculated as mAP_50/_FLOPs, representing the accuracy achieved per unit of computational cost. RubberFormer achieves an efficiency score of 1.15, which is significantly higher than heavyweight models like RT-DETR-X (0.38) and DINO (0.35), while also outperforming them in absolute accuracy. Although lightweight models like YOLOv8 and YOLOv12 achieve higher efficiency scores due to their extremely low FLOPs, RubberFormer surpasses them by 5.4% and 5.2% in mAP_50_, respectively.

The GPU memory consumption of RubberFormer is only 3.2 GB, which is 56.2% lower than RT-DETR-X (7.3 GB) and 48.4% lower than DINO (6.2 GB). The inference latency of 19.7 ms on an RTX 3090 corresponds to approximately 50.8 FPS, which is promising for near-real-time disease monitoring workflows. However, it is important to note that these benchmarks were obtained on a high-performance desktop GPU. Direct deployment on resource-constrained edge devices (e.g., NVIDIA Jetson Orin Nano, Raspberry Pi) would require additional optimization steps such as TensorRT acceleration, INT8 quantization, or knowledge distillation. Preliminary tests using TensorRT FP16 optimization on an NVIDIA Jetson AGX Orin yielded an inference latency of approximately 58 ms (˜17 FPS) for RubberFormer, which is adequate for periodic monitoring (e.g., capturing and analyzing images every few seconds) but falls short of strict real-time video processing requirements (≥30 FPS). Comprehensive edge deployment benchmarks, including INT8 quantization and model pruning, are planned as future work. Nonetheless, the relatively low memory footprint (3.2 GB) and moderate FLOPs (76.6G) position RubberFormer as a strong candidate for GPU-equipped edge platforms commonly used in modern agricultural monitoring systems.

#### 4.3.6 Robustness analysis under challenging conditions

Real-world agricultural environments present numerous challenges that can significantly impact detection performance. Field conditions are inherently variable, with factors such as changing weather, time of day, camera motion, and physical obstructions all affecting image quality. To ensure that RubberFormer can reliably operate under these diverse conditions, we conducted a series of comprehensive stress tests on PM-Dataset-Plus. Specifically, the test images for this analysis were generated by applying synthetic perturbations to the original test set (977 images) using standardized image corruption functions from the Python Albumentations library (Buslaev et al., 2020): illumination variations were simulated by adjusting brightness by ±50%; blur was introduced via Gaussian filtering (*σ* = 1.5) and motion blur (kernel size=7); occlusion was simulated by randomly masking 20% or 40% of bounding box regions with gray patches. These tests systematically evaluate the model’s robustness under three categories of challenging conditions: varying illumination, image quality degradation, and occlusion scenarios. **Table 10** summarizes the comparative results.

**Table 10:** Robustness analysis of RubberFormer under challenging conditions on PM-Dataset-Plus.

Condition	RubberFormer	RT-DETR-X	YOLOv12	Deformable DETR	Δ (Ours vs. Best)
Illumination Variations (mAP_50_%)					
Normal lighting	88.3	85.1	83.1	83.5	+3.2
Low light (-50%)	82.7	78.3	75.6	76.2	+4.4
Overexposure (+50%)	84.1	79.8	77.2	78.5	+4.3
Image Quality Degradation (mAP_50_%)					
Original quality	88.3	85.1	83.1	83.5	+3.2
Gaussian blur (*σ* = 1.5)	83.5	78.9	76.3	77.1	+4.6
Motion blur (kernel=7)	81.2	76.5	73.8	74.6	+4.7
Occlusion Scenarios (mAP_50_%)					
No occlusion	88.3	85.1	83.1	83.5	+3.2
Partial occlusion (20%)	84.6	80.2	77.8	78.9	+4.4
Heavy occlusion (40%)	78.3	73.1	70.5	71.8	+5.2

The robustness analysis reveals that RubberFormer consistently outperforms other methods across all challenging conditions. Under low light conditions, RubberFormer maintains 82.7% mAP_50_, which is 4.4% higher than the second-best method (RT-DETR-X at 78.3%). This superior performance under varying illumination can be attributed to the HALO module’s ability to adaptively fuse features at multiple scales, which helps preserve discriminative information even when image quality is degraded.

For image quality degradation scenarios, RubberFormer demonstrates remarkable resilience. Under Gaussian blur with *σ* = 1.5, the model achieves 83.5% mAP_50_, representing only a 4.8% drop from the original performance. In contrast, YOLOv12 experiences a 6.8% performance drop under the same conditions. The motion blur scenario shows similar trends, with RubberFormer maintaining a 4.7% advantage over the second-best method.

The occlusion analysis is particularly relevant for real-world applications where leaves may be partially obscured by branches, other leaves, or environmental factors. Under heavy occlusion (40%), RubberFormer achieves 78.3% mAP_50_, which is 5.2% higher than RT-DETR-X. This robustness to occlusion is largely due to the UCAN module’s cross-dimensional attention mechanism, which enables the model to leverage contextual information from unoccluded regions to infer the presence and location of partially visible lesions.

#### 4.3.7 Discussion

The experimental results presented in this study demonstrate that RubberFormer achieves state-of-the-art performance in rubber tree powdery mildew detection while maintaining computational efficiency suitable for practical deployment. Through comprehensive evaluation across multiple datasets and experimental conditions, several key findings emerge that provide valuable insights for both the current application and future research directions.

The core contributions of each proposed module can be summarized as follows:

1. The integration of MobileNetV4 as the backbone network proves to be an effective strategy for balancing accuracy and efficiency. While the lightweight backbone alone results in a slight performance drop compared to heavier alternatives, the subsequent addition of HALO and UCAN modules more than compensates for this reduction, ultimately achieving superior accuracy with significantly lower computational cost. This design philosophy aligns with the practical requirements of agricultural monitoring systems, where computational resources are often limited.
2. The HALO module’s hierarchical attention mechanism demonstrates particular effectiveness in handling the multiscale nature of powdery mildew lesions. The module’s ability to adaptively fuse local and global features enables robust detection across different severity levels, from large early-stage lesions to small, fragmented late-stage manifestations. This capability is crucial for comprehensive disease assessment, as accurate detection across all severity levels is essential for timely intervention and treatment planning.
3. The UCAN module’s cross-dimensional attention mechanism addresses a critical limitation of traditional attention mechanisms by enabling collaborative modeling of spatial, channel, and frequency information. This comprehensive feature interaction is especially beneficial for detecting lesions with subtle texture differences from healthy leaf tissue. The synergy between spatial and channel attention allows the model to focus on disease-relevant features while suppressing background noise.
4. The incorporation of NWD Loss significantly improves the localization accuracy for small objects. Unlike traditional IoU-based losses that struggle with tiny targets, NWD Loss provides stable gradients even when predicted and ground-truth boxes have minimal overlap, leading to more accurate bounding box regression for small lesions. This improvement is particularly important for early-stage disease detection, where lesions are typically small and early intervention can prevent significant crop damage.

Beyond the individual module contributions, the generalization capability demonstrated on the PD-40 dataset further validates the transferability of RubberFormer to other plant disease detection tasks. The consistent performance improvement across different crops and disease types suggests that the proposed architectural innovations are not specific to rubber tree powdery mildew but represent general advances in agricultural disease detection. This broad applicability enhances the practical value of the proposed framework for diverse agricultural monitoring scenarios. However, it should be noted that PD-40 was also constructed by researchers affiliated with our group (Li et al., 2025), which means there may be potential overlap in data collection methodology, annotation conventions, and annotator habits. While PD-40 covers 8 different crops and 40 disease categories—providing substantial diversity—we acknowledge this as a limitation. Validation on a fully independent third-party dataset, such as PlantDoc (Singh et al., 2020) adapted for detection tasks, would provide stronger evidence of generalization and is planned as future work.

To provide a balanced assessment of the proposed method, we also analyzed typical failure cases observed during evaluation on PM-Dataset-Plus. The main categories of errors include: (1) *False positives* caused by visually similar artifacts such as water droplets, dust deposits, or insect damage marks being misidentified as powdery mildew lesions, particularly at early infection stages where the visual distinction is subtle; (2) *False negatives* occurring primarily at severe infection levels (Level 7–9), where densely clustered tiny lesions are partially merged or missed due to overlapping bounding box suppression during NMS; and (3) *Duplicate detections*, where multiple overlapping bounding boxes are generated for a single lesion region, especially for larger irregularly-shaped lesions spanning Level 3–5. Among all test images, approximately 3.8% of detection errors were attributable to false positives, 5.1% to missed detections, and 2.3% to duplicate boxes. To address these issues in future work, we plan to incorporate context-aware NMS strategies, explore soft-NMS (Bodla et al., 2017) for reducing missed detections in dense scenes, and integrate domain-specific post-processing rules that leverage lesion shape priors to filter false positives.

Despite these achievements, several limitations should be acknowledged for future improvement. The current framework is designed for single-image detection and does not exploit temporal information that could be valuable for disease progression monitoring. Incorporating temporal modeling could enable more accurate prediction of disease development trends. Additionally, while the model achieves promising inference speed on GPU-equipped devices, the FPS benchmarks reported in this study were measured on a high-performance RTX 3090 GPU. As discussed in Section 4.3.5, preliminary tests on an NVIDIA Jetson AGX Orin showed reduced throughput (˜17 FPS), and further optimization through techniques such as model quantization, pruning, and knowledge distillation will be necessary for deployment on more resource-constrained edge devices. Future work will also explore the integration of multi-modal data sources, such as hyperspectral imaging and environmental sensors, to further enhance detection accuracy and enable more comprehensive disease assessment.

## 5 Conclusion

To address the challenge of accurately and efficiently detecting rubber tree powdery mildew in complex field environments, this study proposes a novel end-to-end detection framework called RubberFormer. The framework integrates a lightweight MobileNetV4 backbone, a HALO module for enhanced multiscale feature perception, and a UCAN module for improved cross-dimensional feature interaction. Additionally, the introduction of NWD Loss into the localization loss significantly improves the detection of small and irregular lesions. Comprehensive experimental results on both the self-constructed large-scale PM-DatasetPlus and the PD-40 dataset demonstrate that RubberFormer maintains a strong balance between detection accuracy, inference speed, and model complexity, consistently surpassing mainstream detection methods.

This study not only presents a high-performance and deployable technical solution for the automated monitoring of rubber tree powdery mildew but also provides essential support for early warning and precision control of the disease. More importantly, the design principles of RubberFormer, which focus on balancing model efficiency and detection performance, offer valuable insights for other agricultural vision tasks involving small objects and complex backgrounds. Future work will focus on further improving deployment efficiency on edge devices, exploring dynamic disease prediction using time-series modeling, and extending the framework to broader crop disease monitoring applications, contributing to the standardization and intelligent development of smart agriculture.

## Data Availability

The datasets presented in this study are available in online repositories. PM-Dataset-Plus is available at https://github.com/wfcyliyuheng-dev/PM-Dataset-Plus. PD-40 is available at https://github.com/wfcyliyuheng-dev/PD40-Dataset.
